# Predictors of Transphobia and Attitudes Toward Transgender Individuals Among Nurses in Türkiye: A Cross-Sectional Study

**DOI:** 10.3390/healthcare13121474

**Published:** 2025-06-19

**Authors:** Ezgi Şişman, Mehtap Güngör, Aila Gareayaghi, Hanife Yılmaz, Aslıhan Polat

**Affiliations:** 1 Department of Psychiatry, Faculty of Medicine, Kocaeli University, İzmit 41001, Kocaeli, Türkiye; 2 Psychiatry Clinic, Kocaeli City Hospital, İzmit 41060, Kocaeli, Türkiye; 3 Şemdinli State Hospital, Şemdinli 30800, Hakkari, Türkiye; 4 Community Mental Health Center, Kocaeli University, İzmit 41001, Kocaeli, Türkiye

**Keywords:** transphobia, transgender persons, nursing attitudes, predictive factors, cultural competence, transgender health education, Turkey

## Abstract

**Background/Objectives:** Transgender individuals face significant healthcare disparities, often exacerbated by provider prejudice and knowledge gaps. Nurses, as frontline providers, play a critical role in promoting inclusive care. This study aimed to evaluate the levels and predictors of transphobia and attitudes toward transgender individuals among nurses in Türkiye. **Methods**: A cross-sectional survey was conducted with 100 registered nurses. Participants completed the Transphobia Scale, the Attitudes Toward Transgender Individuals Scale, and the Hudson & Ricketts Homophobia Scale. Additional measures included perceived knowledge, prior education on transgender health, familial attitudes, and comfort levels when working with transgender patients. Multiple linear regression analyses identified predictors of transphobia and attitudes. **Results**: The mean Transphobia Scale score was 41.53 (SD = 12.67), and the mean Attitudes Toward Transgender Individuals Scale score was 57.45 (SD = 16.42). Greater homophobia, negative parental attitudes, and higher perceived knowledge significantly predicted higher transphobia scores (Adjusted R^2^= 0.327). Prior education on transgender health was also associated with lower transphobia. Lower comfort with transgender patients was marginally associated with higher transphobia. More positive attitudes toward transgender individuals were predicted by lower transphobia, lower homophobia, and prior education (Adjusted R^2^ = 0.526). **Conclusions**: Findings underscore the urgent need for structured transgender health education and culturally sensitive interventions among nurses. Addressing sociocultural factors and training gaps could enhance equitable healthcare delivery for transgender populations, particularly in culturally conservative settings like Türkiye.

## 1. Introduction

### 1.1. Transgender Individuals and Structural Barriers in Healthcare Access

Transgender individuals face profound health disparities, largely due to systemic barriers that hinder access to equitable healthcare. These barriers include stigma, discrimination, socio-economic exclusion, and disproportionately high rates of violence, including sexual victimization compared to their cisgender peers [1,2]. As a marginalized and often overlooked gender group, transgender people encounter widespread exclusion from healthcare systems [3]. Discrimination within healthcare settings has been documented globally and is recognized as a significant contributor to negative health outcomes among transgender populations. Evidence suggests that these challenges are not isolated incidents but reflect systemic shortcomings in the delivery of care to transgender individuals [3].

Transgender, gender non-conforming, and non-binary individuals frequently report disrespect and discrimination by healthcare providers, which often leads them to avoid or postpone seeking care, even when necessary [4]. These challenges are multifaceted and include financial barriers, provider bias, and a general lack of culturally competent services [5]. Experiences such as being denied care, misgendered, or subjected to verbal, physical, or sexual abuse within medical settings further exacerbate health disparities [6]. Delayed care due to anticipated discrimination is a well-documented public health concern and is associated with both poorer physical and mental health outcomes [7]. Even in countries with advanced human rights protections, transgender individuals continue to experience widespread healthcare inequities [8,9]. 

These systemic shortcomings are not unique to healthcare but rather mirror broader patterns of sociopolitical exclusion, especially in societies where patriarchal and heteronormative norms are deeply institutionalized. In countries like Türkiye, gender and sexual minorities are routinely portrayed as threats to national values [10,11]. In such a climate, healthcare discrimination becomes a natural extension of this wider marginalization.

### 1.2. The Role of Healthcare Providers in Transgender Health Inequities

Transphobia within healthcare settings extends beyond individual prejudice to reflect systemic and institutional failures that compromise the quality and accessibility of care for transgender individuals. A growing body of literature highlights that healthcare professionals—whether through implicit bias, lack of training, or overt discriminatory behavior—play a critical role in reinforcing stigma and exclusion [6,12]. These dynamics contribute to healthcare avoidance, particularly when transgender patients anticipate misgendering, insensitive language, or denial of care [13,14,15].

Miscommunication, professional distancing, and the use of cisnormative assumptions further exacerbate exclusion, often placing the burden of education on transgender patients themselves [14,16]. These experiences not only compromise trust in the provider–patient relationship but also perpetuate negative health outcomes across physical and mental domains [17,18]. Moreover, transphobia in clinical settings often extends beyond attitudinal bias, encompassing systemic and affective forms of harm embedded in medical interactions. Atuk conceptualizes such harm as “iatrogenic,” illustrating how trans bodies are marked as untouchable or suspicious in Turkish healthcare encounters—where clinical intimacy can become a site of symbolic and physical violence [19]. In contrast, professionals who receive adequate training and demonstrate cultural competence are more likely to deliver inclusive, affirming, and person-centered care [20].

Sociodemographic variables such as gender identity, openness to experience, and feminist orientation have also been shown to influence attitudes, with women and non-binary individuals generally reporting greater empathy toward transgender patients [21,22]. However, many healthcare workers continue to feel ill-equipped to meet the needs of transgender patients, reflecting persistent gaps in education and training [14,23].

### 1.3. Why Nurses Matter: Their Pivotal Role in Transgender-Inclusive Healthcare

As frontline healthcare providers, nurses play a critical role in either facilitating or hindering access to gender-affirming care. Transgender patients frequently encounter discrimination from all levels of healthcare staff, including nurses, who are often the first point of contact [24]. When nurses lack the necessary knowledge or harbor prejudiced attitudes, it can directly affect the quality of care and perpetuate marginalization [25,26]. Negative experiences with nurses contribute to mistrust, nondisclosure, and healthcare avoidance among transgender individuals [15,27].

### 1.4. Knowledge Gaps as a Driver of Transphobia in Healthcare Settings

Across disciplines, limited education has emerged as a consistent determinant of transphobic attitudes [14,28] Among healthcare providers, inadequate knowledge about gender identity, terminology, and health needs results in uncertainty and discomfort, which may in turn foster exclusionary behaviors [23]. Educational interventions that merely raise awareness often fall short; experiential learning and structured clinical exposure are more likely to produce durable attitudinal shifts [18,29].

Within nursing education specifically, curricula often omit transgender-specific content, leaving future professionals underprepared to provide competent, inclusive care [30]. Students frequently report confusion over gender-related concepts and endorse heteronormative assumptions, emphasizing the urgent need for curriculum-integrated, competency-based instruction [31]. A lack of simulation-based or experiential learning opportunities further limits their preparedness [29]. Inclusive education and practical training are essential for promoting empathy and reducing discriminatory behavior [18].

### 1.5. Transgender Healthcare Education and Research in Türkiye: A Neglected and Increasingly Contested Domain

Transgender healthcare education in Türkiye remains severely underdeveloped despite growing international attention to the healthcare needs of gender-diverse populations. Local studies echo global findings, documenting discriminatory behavior by healthcare providers, including treatment refusal, prolonged waiting periods, and an overall lack of transgender health literacy [32,33]. While international clinical guidelines for gender-affirming care have been well established, these recommendations have not been systematically integrated into formal healthcare education in Türkiye [34,35].

Educational activities related to transgender health are sporadic, typically restricted to limited sessions within psychiatric or sexuality-themed congresses. This may partially explain the comparatively lower levels of transphobia reported among psychiatric professionals relative to other healthcare providers [36]. However, structural limitations on research and training have intensified in recent years amid an increasingly hostile sociopolitical climate.

Several faculties of medicine known for providing gender-affirming care have been publicly targeted through political and media channels, with some facing legal investigations for their involvement in transgender health services [1,10]. Furthermore, rising anti-LGBTQ+ discourse—exemplified by parliamentary efforts to introduce punitive legal measures and mass public demonstrations framed as “family marches”—has created a chilling effect across academic and healthcare institutions [10,37]. In the absence of comprehensive national data on LGBTQ+ health and amidst mounting societal stigma, transgender individuals remain underrepresented in healthcare settings, and research in this area is often discouraged or politicized. As a result, these dynamics collectively constrain the development of inclusive health policies, impede evidence-based educational reforms, and limit the visibility of transgender health issues in academic discourse.

These pressures have contributed to the discontinuation or downsizing of clinical units offering transgender-specific services, thereby undermining the already scarce opportunities for practical training and multidisciplinary exposure. These structural and ideological barriers not only hinder the integration of transgender health education into mainstream medical and nursing curricula but also limit research production and access to clinical learning environments. As a result, the training of future healthcare professionals in transgender-specific care remains fragmented, institution-dependent, and vulnerable to political shifts. This institutional vulnerability not only reflects logistical limitations but also deeper affective and regulatory dynamics embedded within the healthcare system. Recent ethnographic scholarship emphasizes that transphobia in healthcare settings cannot be fully understood without examining how institutions mobilize intimacy as a site of control. Zengin conceptualizes medical and bureaucratic interventions on trans bodies as “violent intimacies,” wherein acts of care, diagnosis, or examination are entangled with practices of regulation, surveillance, and affective violence [38]. In such a climate, even small-scale educational initiatives acquire heightened significance, serving as rare yet critical opportunities to develop professional competence and mitigate systemic neglect.

### 1.6. Theoretical Framework: The Minority Stress Model

In understanding the origins and manifestations of provider prejudice, the Minority Stress Model offers a valuable framework [39]. The model posits that stigma functions as a distal stressor that becomes internalized over time, shaping attitudes and behaviors through socialization processes. Within healthcare, such mechanisms may influence not only the experiences of patients but also the emotional and cognitive dispositions of care providers. Constructs such as familial attitudes, comfort levels, and exposure to structured education—examined in this study—may function as mediators or moderators within this theoretical framework.

Although the relationship between knowledge, experience, and bias has been explored globally, there are limited empirical data from culturally conservative health systems. This study addresses this gap using data from Türkiye.

### 1.7. Aim of the Study

This study aims to evaluate the attitudes and levels of transphobia among nurses working at a university hospital in Türkiye. Beyond assessing baseline knowledge and identifying training gaps, the study investigates the sociodemographic, educational, experiential, and sociocultural predictors of both transphobia and attitudes toward transgender individuals in clinical settings.

To guide the analysis, the study was informed by several conceptually grounded expectations. Individual factors such as homophobia, perceived and objective knowledge, and comfort in working with transgender individuals were hypothesized to be associated with levels of transphobia and general attitudes. Socialization-related variables, including parental attitudes and religious beliefs, were considered relevant predictors given their role in shaping value systems. Educational exposure—specifically prior training on transgender health—was expected to support more inclusive perspectives. Finally, transphobia itself was examined not only as a dependent variable but also as a potential predictor of overall attitudinal orientation toward transgender individuals, in line with theoretical models linking affective bias to behavioral intention. 

## 2. Materials and Methods

### 2.1. Design

This cross-sectional study was conducted with 100 nurses employed at a tertiary-level university hospital in Türkiye between December 2022 and February 2023. Nurses from various departments, including surgical, internal medicine, emergency, and psychiatry units, were eligible to participate. The study was conducted in collaboration with the hospital’s Nursing Directorate, as part of an ongoing institutional collaboration led by the psychiatry department. The Directorate facilitated the random selection process and endorsed the distribution of questionnaires in line with the broader goals of educational development within the hospital. 

### 2.2. Setting, Sampling, Inclusion/Exclusion Criteria

Inclusion criteria included being a currently employed registered nurse providing direct patient care. Nurses who had previously participated in similar surveys or submitted incomplete responses were excluded. As part of an upcoming institutional in-service training program aimed at improving equitable care for transgender individuals, the program was structured into five sessions: the first four were designed for groups of 100 nurses, and the final session was planned to accommodate 80 participants. To inform the content of these sessions, the hospital nursing directorate was asked to randomly select 100 nurses from the pool of eligible staff. Accordingly, these selected individuals were invited to voluntarily participate in the study. They were informed that participation was entirely optional, that all responses would remain anonymous, and that their answers would have no impact on their professional standing or licensure status.

The nursing directorate distributed paper-based, self-administered questionnaires to the selected nurses in sealed envelopes. Participants completed the surveys privately at home and returned the sealed envelopes to the nursing office, where they were anonymously collected in a locked drop-box. No identifying information was collected at any stage, and all 100 surveys were returned fully completed, yielding a response rate of 100%.

A formal a priori power analysis was not conducted, as the final number of predictors (n = 12) was determined based on exploratory bivariate analyses. However, a post hoc power analysis using G*Power 3.1 indicated that the sample size was sufficient to detect a medium effect size (f^2^ = 0.15) with 12 predictors at α = 0.05 and power (1 − β) = 0.80. Considering the sociopolitical sensitivity of the topic in Türkiye, the achieved sample size was deemed both statistically sufficient and contextually meaningful.

### 2.3. Instruments

#### 2.3.1. Demographic and Professional Characteristics

A personal information form was used to collect data on age, gender, level of education, years of professional experience, department, whether the participant had previously worked with a transgender person, and whether they had received any training on transgender issues. Familial attitudes toward transgender individuals were assessed via a single self-report item asking how such topics were approached in the participant’s family environment (supportive, neutral, or negative). This reflects the affective tone of early socialization around gender and identity. Comfort levels were measured by asking participants how comfortable they felt interacting with transgender, LGB, and intersex patients and colleagues, using a 5-point Likert scale (1 = very uncomfortable, 5 = very comfortable). These items aimed to capture affective readiness in clinical or team-based contexts. While single-item measures may limit psychometric precision, they are frequently used to capture affective readiness in large-scale attitudinal studies and were deemed contextually appropriate for this exploratory design.

#### 2.3.2. Knowledge Assessment

Participants’ conceptual knowledge was assessed using six multiple-choice questions specifically developed for this study. Each item was designed to evaluate the participant’s ability to correctly identify key constructs, including gender identity, biological sex, gender dysphoria, heterosexuality, homosexuality, and bisexuality. A composite knowledge score was calculated as the total number of correct responses, with possible scores ranging from 0 to 6. The transgender knowledge questions were developed by the research team based on key conceptual distinctions in transgender health literature. To ensure content clarity and contextual relevance, the items were reviewed by the academic coordinator of the university’s psychiatric nursing department, the physician responsible for the transgender outpatient unit, and unit-affiliated nursing staff, all of whom have clinical experience with gender-affirming care. While these six items were reviewed for face and content validity by clinicians and educators experienced in transgender health, they were not subjected to psychometric validation and should be interpreted as exploratory measures of basic conceptual understanding. In addition to objective knowledge, perceived knowledge was assessed through self-rated familiarity with transgender, LGB, and intersex health on a 5-point Likert scale (1 = no knowledge, 5 = expert level). This variable captures subjective confidence rather than tested competence, in line with previous operationalizations [40].

#### 2.3.3. Attitudes Toward Transgender Individuals

The Attitudes Toward Transgender Individuals Scale, developed by Walch et al., was used to assess participants’ overall attitudes [41]. The scale consists of 20 items rated on a 5-point Likert scale (1 = strongly disagree to 5 = strongly agree). Higher scores indicate more positive attitudes toward transgender individuals. The Turkish version of the scale has demonstrated strong reliability, with a Cronbach’s alpha coefficient of 0.96 [42]. In this study, the total score ranged from 20 to 100, with higher scores reflecting more favorable attitudes.

#### 2.3.4. Transphobia Scale

The Transphobia Scale developed by Nagoshi et al. was used to evaluate participants’ level of discomfort or prejudice toward transgender people [43]. The scale includes 9 items rated on a 7-point Likert scale (1 = strongly disagree to 7 = strongly agree). Higher scores indicate greater levels of transphobia. The Turkish version of the scale was adapted using the back-translation method and pilot-tested among bilingual individuals, demonstrating excellent test–retest reliability (rs = 0.98, *p* < 0.01) and internal consistency (Cronbach’s alpha = 0.82) [44]. In this study, the possible total score ranged from 9 to 63, with higher scores reflecting greater levels of transphobia.

#### 2.3.5. Homophobia Scale

Homophobia was assessed using the Hudson and Ricketts Homophobia Scale, a 25-item instrument originally developed to measure negative attitudes toward homosexual individuals [45]. In this study, the 24-item Turkish version adapted by Sakallı and Uğurlu was utilized [46]. This version has demonstrated excellent internal consistency in previous research (Cronbach’s alpha = 0.94). Participants were asked to rate each item on a 6-point Likert scale ranging from 1 (strongly disagree) to 6 (strongly agree), with higher scores indicating greater levels of homophobia. The total score thus ranged from 24 to 144. In the current study, only the total score was used, and it served as the primary outcome variable in subsequent analyses.

### 2.4. Statistical Analysis

All statistical analyses were conducted using IBM SPSS (Statistical Package for the Social Sciences) for Windows, version 22.0. Descriptive statistics were used to summarize the sample characteristics and main variables. Normality was assessed using the Kolmogorov–Smirnov test due to the sample size (N = 100). As most variables were not normally distributed, non-parametric tests (Mann–Whitney U and Kruskal–Wallis) were employed for group comparisons, and Spearman’s correlation coefficients were calculated to examine associations between continuous or ordinal variables. For variables with more than two categories that showed statistically significant group differences (e.g., education level), post-hoc pairwise comparisons were conducted using Bonferroni-adjusted Mann–Whitney U tests to determine the specific group differences. Although non-parametric methods were applied for bivariate analyses, the dependent variables—transphobia and attitudes toward transgender individuals—demonstrated approximately normal distributions based on Shapiro–Wilk tests (*p* = 0.211 and *p* = 0.041, respectively), skewness values (0.21 and 0.27), and histogram inspection. Therefore, the assumptions required for linear regression were considered sufficiently met. Multiple linear regression models were constructed to identify predictors of transphobia and attitudes toward transgender individuals. In building these models, priority was given to variables that were statistically significant in group comparisons or correlation analyses with the dependent variable, as well as those considered clinically relevant or supported by the literature. Multicollinearity was assessed by calculating variance inflation factors (VIFs). All VIF values remained below the accepted threshold of 5, indicating no significant multicollinearity among predictors. Statistical significance was set at *p* < 0.05.

### 2.5. Ethical Considerations

Ethical approval for the study was obtained from the Kocaeli University Non-Interventional Clinical Research Ethics Committee (2019/KUGOEK01.4).

## 3. Results

As shown in Table 1, the sample consisted predominantly of female nurses (93.0%), with a mean age of 29.86 years (SD = 5.98) and a mean professional experience of 8.04 years (SD = 5.76). Most participants were married (59.0%) and held a bachelor’s degree (63.0%). Only 7.0% reported prior work experience in a psychiatric setting. Nearly all participants identified as heterosexual (99.0%), and most reported residing in urban areas (92.0%).

Regarding religious orientation, 90.0% of participants reported believing in a religion and attempting to practice it, 5.0% reported fully practicing all religious requirements, 3.0% did not believe in God, and 2.0% believed in God but not in religion. For descriptive clarity, a more detailed item on religious orientation (with four response options) is presented in the narrative, although a three-category version was used for all statistical analyses and is displayed in Table 1.

A total of 21.0% reported knowing an LGB individual, and 7.0% knew a transgender individual personally. Nineteen percent had encountered a transgender person in a professional context. Within the family setting, 46.0% indicated that sexuality was never discussed, 37.0% reported it was rarely discussed, and 17.0% reported occasional discussions. Homosexuality and transgender-related issues were discussed in 20.0% and 17.0% of families, respectively. Parental attitudes toward transgender individuals were originally assessed with three response options: positive, neutral, and negative. Notably, no participants in the current sample selected the ‘positive’ option.

Self-rated knowledge levels varied across sexual and gender minority health topics. Twenty-five percent reported no knowledge of LGB health, 31.0% no knowledge of transgender health, and 61.0% no knowledge of intersex health. Good knowledge of intersex health was reported by 2.0%. Regarding conceptual distinctions, 13.0% reported a good understanding of the difference between gender identity and sexual orientation, and 12.0% for gender identity and biological sex. One percent identified themselves as experts in the latter. 

In terms of comfort, 37.0% of participants reported feeling comfortable or very comfortable working with LGB patients, 38.0% with transgender patients, and 29.0% with intersex patients. Comfort with LGBTI colleagues was reported by 33.0%. Among participants who reported comfort working with LGB patients, 78.4% also reported comfort working with LGBTI colleagues. Similar patterns were observed among those comfortable working with transgender (76.3%) and intersex patients (75.9%).

Conceptual knowledge items revealed that 83.0% of participants correctly identified the definitions of “gender identity” and “biological sex”. Correct identification rates were 39.0% for “gender dysphoria,” 73.0% for “bisexuality,” 87.0% for “heterosexuality,” and 86.0% for “homosexuality”. A detailed overview of all descriptive characteristics is provided in Table 1.

Table 2 presents group comparisons of transphobia and attitudes toward transgender individuals across categorical variables. Statistically significant differences in transphobia scores were observed according to education level (*p* = 0.015), discussion of transgender topics within the family (*p* = 0.020), parental attitudes toward transgender individuals (*p* = 0.014), and receiving institutional training on transgender issues (*p* = 0.039). Post hoc analyses revealed that nurses with a bachelor’s degree reported significantly higher transphobia scores than both high school graduates (*p* = 0.024) and those with a master’s degree or higher (*p* = 0.008). No significant difference was observed between high school and master’s+ groups (*p* = 0.785). Participants who had received transgender-related education reported significantly lower transphobia scores than those who had not. Moreover, greater comfort working with transgender patients (*p* = 0.007) and LGBTI colleagues (*p* = 0.001) was associated with lower levels of transphobia. 

Attitudes toward transgender individuals also differed significantly by marital status (*p* = 0.005), parental attitudes toward transgender individuals (*p* = 0.047), beliefs about transgender identity (*p* = 0.019), and education-related variables. Notably, those who had received education on transgender health (*p* < 0.001) or expressed willingness to receive such training (*p* = 0.002) reported more positive attitudes. Similarly, greater self-reported comfort working with transgender patients (*p* < 0.001) and LGBTI colleagues (*p* < 0.001) was strongly associated with more favorable attitudes. 

Significant bivariate associations were observed between transphobia and several psychosocial variables, as detailed in Table 3. Transphobia was negatively correlated with attitudes toward transgender individuals (r = −0.54, *p* < 0.01) and positively correlated with homophobia (r = 0.55, *p* < 0.01) and negative parental attitudes (r = 0.25, *p* < 0.05). Additional significant correlations included lower comfort working with transgender patients (r = −0.35, *p* < 0.01) and lack of prior education on transgender health (r = −0.31, *p* < 0.01). In terms of attitudinal outcomes, attitudes toward transgender individuals were negatively associated with homophobia (r = −0.65, *p* < 0.01), age (r = −0.26, *p* < 0.01), and parental negativity (r = −0.20, *p* < 0.05) and positively associated with comfort working with transgender patients (r = 0.43, *p* < 0.01) and prior transgender health education (r = 0.38, *p* < 0.01).

Multivariate linear regression analysis revealed several significant predictors of transphobia scores. Higher levels of perceived knowledge (ß = 0.245, *p* = 0.011) and homophobia (ß = 0.394, *p* < 0.001) were positively associated with transphobia. Receiving education on transgender health was significantly associated with lower transphobia levels (ß = 0.254, *p* = 0.010), while negative parental attitudes also contributed significantly to increased transphobia (ß = −0.223, *p* = 0.026). Additionally, lower comfort when working with transgender patients was marginally associated with higher transphobia scores (ß = −0.191, *p* = 0.058). The overall model was statistically significant, F(8, 91) = 7.026, *p* < 0.001, explaining 32.7% of the variance in transphobia scores (Adjusted R^2^ = 0.327), as presented in Table 4.

Table 5 presents the results of the multivariate linear regression analysis examining predictors of attitudes toward transgender individuals. Receiving education on transgender health (ß = 0.195, *p* = 0.016), lower levels of homophobia (ß = −0.245, *p* = 0.009), and lower levels of transphobia (ß = −0.277, *p* = 0.003) were significantly associated with more positive attitudes. The overall model was statistically significant, F(13, 86) = 10.14, *p* < 0.001, explaining 52.6% of the variance in attitudes (Adjusted R^2^ = 0.526).

## 4. Discussion

This study aimed to identify predictors of transphobia and attitudes toward transgender individuals among nurses working in a university hospital in Türkiye. Two separate multivariate linear regression models were constructed to evaluate the associations of various demographic, experiential, and psychosocial factors with transphobia and attitudes. The findings indicate that higher levels of homophobia, increased perceived knowledge, negative parental attitudes, and lower comfort in working with transgender individuals were associated with greater transphobia. Conversely, receiving prior education on transgender health was associated with lower transphobia scores. Comfort working with transgender patients showed a marginal association with transphobia, indicating a potential effect that warrants further investigation. In the second model, more positive attitudes toward transgender individuals were significantly predicted by lower transphobia and homophobia levels, as well as prior education on transgender health. These results suggest that both cognitive-affective variables and prior training experiences play a critical role in shaping nurses’ beliefs and dispositions toward transgender individuals.

Among the significant predictors, homophobia showed the strongest statistical association with transphobia. This finding is consistent with previous research that reported strong correlations between general sexual prejudice and specific biases toward transgender individuals [31,47]. Therefore, including homophobia as a theoretically important predictor was essential to better isolate attitudinal factors specific to transgender issues.

Interestingly, perceived knowledge showed a positive association with transphobia, suggesting that self-reported confidence may not align with inclusive attitudes. Although perceived knowledge was not significantly correlated with transphobia in bivariate analysis, it emerged as a significant predictor in the multivariate regression model. This discrepancy may reflect a potential suppressor effect, where the variable’s unique contribution becomes apparent only when modeled alongside others. We acknowledge this complexity and believe that the multivariate approach offers a more accurate representation of the underlying relationships. This challenges the assumption that all forms of knowledge reduce prejudice and may reflect overconfidence or superficial familiarity in the absence of formal education. While knowledge is often expected to reduce prejudice, some studies have noted that perceived knowledge—particularly when not supported by structured training—can foster overconfidence and reinforce existing biases. For instance, Stroumsa et al. emphasize that actual transgender health knowledge, rather than self-perceived knowledge, predicts clinical competence, suggesting that superficial familiarity may hinder rather than help inclusive care efforts [40].

Consistent with existing literature, prior education on transgender health was a significant protective factor in both regression models, predicting lower transphobia and more positive attitudes. This aligns with findings from a recent systematic review by Jecke and Zepf, which underscored the positive impact of transgender-specific training on professional attitudes [48]. The consistency across studies underscores the utility of integrating structured, evidence-based content into continuing education and undergraduate nursing curricula. Importantly, the protective effect of education remained statistically significant after adjusting for other variables such as perceived knowledge and comfort, indicating a consistent trend across both models.

While prior education on transgender health has been identified as a protective factor against transphobia and negative attitudes among healthcare professionals, the current sociopolitical climate in Türkiye presents significant challenges to such educational initiatives. There has been an observable increase in hostile rhetoric and actions targeting LGBTQ+ individuals, including healthcare professionals working in gender dysphoria clinics. Notably, some media narratives have publicly stigmatized clinicians providing care in this field, contributing to an atmosphere of fear and institutional withdrawal [10]. Although participants who reported receiving institutional education on transgender health exhibited slightly higher mean transphobia scores than those who did not, this counter-intuitive finding was based on a very small subgroup (n = 4). Given the limited sample size, no meaningful interpretation could be derived, and this variable was therefore not included in the final regression model.

This environment has led to the closure of several transgender health units, thereby limiting opportunities for practical training and exposure for medical and nursing students. The reduction in clinical training sites poses a risk to the competency of future healthcare providers in delivering inclusive care to transgender individuals.

Despite these challenges, some institutions continue to uphold their commitment to transgender health education. For example, the Gender Dysphoria Clinic at Kocaeli University remains operational and actively contributes to educational efforts. A recent example includes both a panel presentation and a workshop on transgender patient care featured in the scientific program of the National Psychiatric Nursing Congress, highlighting the potential of continued professional development even under structural constraints [49].

Comfort working with transgender patients showed a marginal association with transphobia, with lower comfort levels tending to predict higher transphobia scores. This finding parallels the work of Fradelos et al., who demonstrated that reduced interpersonal anxiety and increased experiential familiarity contribute to attitudinal improvement [31]. These findings underscore the value of including exposure-based learning and simulation activities in nursing curricula.

A novel finding of this study is the identification of negative parental attitudes as a significant predictor of transphobia, a variable that has rarely been included in prior regression models in this field. Early familial socialization likely shapes moral frameworks and implicit biases that persist into professional life, a notion consistent with Nagoshi et al.’s (2008) emphasis on the role of socially conservative attitudes—such as benevolent sexism and religious fundamentalism—in the development of prejudice against transgender individuals [43]. Our findings also align with the Minority Stress Model, which underscores how social stigma and early familial discourse may contribute to internalized bias [39]. By formally including socio-cultural constructs—such as parental attitudes and religious beliefs—into multivariate models, this study offers a more comprehensive understanding of how internalized belief systems influence provider attitudes. These results emphasize the importance of targeting not only knowledge deficits but also culturally rooted value systems in efforts to reduce transphobia in nursing practice.

While parental attitudes were found to be predictive, other sociocultural variables exhibited more complex patterns. Although religious belief was assessed, it did not emerge as a significant predictor in the final regression model. This may reflect the complexity of how religious identity interacts with professional norms, institutional culture, and individual discretion in the Turkish healthcare context.

In the second regression model, transphobia was a statistically significant predictor of attitudes toward transgender individuals. This supports the hypothesis that general discomfort or fear toward transgender individuals can act as a cognitive filter, negatively shaping even consciously held attitudes. The interconnectedness of prejudice and attitudes has also been noted in García-Acosta et al., where attitudinal ambivalence was often rooted in deeper affective biases [47].

Building on these findings, this study contributes to the growing body of literature exploring nurse attitudes toward transgender individuals in Türkiye by integrating both educational and sociocultural predictors. Future research may build on this by employing longitudinal designs or assessing how such attitudes translate into actual care practices.

### 4.1. Strengths

This study presents several methodological and contextual strengths that enhance its contribution. It is among the few in Türkiye to examine both transphobia and attitudes toward transgender individuals using two distinct but related regression models, enabling a nuanced analysis of attitudinal dynamics. The combination of validated psychometric instruments with clinician-reviewed knowledge items allowed the simultaneous assessment of both objective and perceived knowledge—an approach rarely integrated into a single design. The study also incorporated a diverse range of predictors—including social, affective, and educational dimensions—offering a more multidimensional and culturally grounded understanding of bias. Including sociocultural variables such as familial discourse and religious belief further supported this culturally embedded interpretation. The survey was administered in a way that reduced social desirability bias, with participants completing it privately at home and returning responses anonymously in sealed envelopes, which likely enhanced the reliability of responses by minimizing perceived institutional pressure. Additionally, the study was conducted in collaboration with the hospital’s nursing directorate and embedded in an ongoing institutional training program, increasing its ecological validity and relevance for practice.

### 4.2. Limitations

Several limitations should be noted. The single-center, cross-sectional design limits generalizability and precludes causal inference. Nonetheless, given the scarcity of transgender-focused healthcare services in Türkiye, access to even a single active institution offered a rare but valuable opportunity. While this institutional homogeneity may limit external validity, it enhances internal consistency by reducing site-level confounds. The knowledge items, though reviewed for content validity by experienced clinicians and educators, were not subjected to formal psychometric validation, which limits comparability with standardized measures. Key psychological predictors such as empathy, openness to diversity, and authoritarianism were not assessed despite their known relevance in previous studies [31]. Although the sample size was sufficient for regression analysis, it consisted predominantly of cisgender female nurses, reflecting the national nursing workforce but limiting subgroup analyses. Despite efforts to ensure anonymity—such as allowing participants to complete the survey at home and return it in sealed envelopes—the sensitive nature of the topic may still have introduced some degree of social desirability bias. While the 100% response rate suggests strong institutional engagement, it could also partially reflect impression management tendencies, limiting the generalizability of self-reported attitudes. Some constructs—such as parental attitudes and comfort—were measured using single items. While meaningful, future research would benefit from validated multi-item instruments and psychometric exploration, particularly for self-developed variables used in this exploratory design.

Future research should explore these predictors longitudinally and across different institutional settings. In addition, integrating qualitative data could help illuminate the mechanisms through which education, familial socialization, and clinical exposure shape professional beliefs. Policymakers and curriculum developers are encouraged to design training modules that combine cognitive content with experiential and reflective components to address both knowledge gaps and deep-seated affective biases. In addition to enhancing individual-level training, these findings underscore the urgency of integrating transgender health into institutional policy frameworks and national nursing curricula, ensuring that inclusive care becomes a standardized component of professional practice.

Beyond individual-level training, institutional protocols play a crucial role in operationalizing transgender-inclusive care. For instance, Kocaeli University Medical Faculty has implemented a multidisciplinary protocol for managing gender dysphoria, which is detailed in a nationally published textbook on transgender healthcare [50]. Such structured models exemplify how academic institutions can lead the way in formalizing transgender care pathways within Türkiye’s complex healthcare landscape. Scaling these efforts and incorporating similar content into national nursing curricula could serve as a meaningful step toward reducing systemic disparities and enhancing the competence of future healthcare providers.

## 5. Conclusions

This study demonstrates that transphobia among nurses is shaped not only by knowledge deficits but also by broader emotional and sociocultural dynamics. Homophobia emerged as the strongest predictor, underscoring how general sexual prejudice can affect professional attitudes toward transgender individuals. The unexpected positive link between perceived knowledge and transphobia suggests that confidence, when not supported by structured education, may reinforce bias. In contrast, prior training on transgender health consistently predicted lower transphobia and more inclusive attitudes, affirming its potential role in enhancing clinical competence. Negative parental attitudes and lower comfort in working with transgender patients were also associated with higher transphobia, reflecting the lingering influence of early socialization and the importance of experiential learning. Importantly, transphobia itself predicted more negative attitudes toward transgender individuals, revealing its broader influence on care orientation.

Taken together, these findings point to the value of educational strategies that go beyond technical instruction and incorporate reflective, socially responsive, and ethically aware elements. Exposure-based methods, such as simulation and direct interaction, appear promising for strengthening not only cognitive competence but also affective readiness. Institutional examples—such as the sustained efforts at Kocaeli University—offer insight into how inclusive training can be maintained even under structural constraints. Future research could explore these dynamics through longitudinal and mixed-method designs, while nursing education and policy may benefit from fostering transformative care approaches—anchored not only in knowledge but also in professional integrity and ethical responsibility.

## Figures and Tables

**Table 1:** Sociodemographic, educational, and professional characteristics of the nursing sample.

Categorical Variables	Response	n (%)
Gender	Female	93 (93.0%)
	Male	7 (7.0%)
Marital status	Married	59 (59.0%)
	Single	41 (41.0%)
Education level	High school	13 (13.0%)
	Associate degree	12 (12.0%)
	Bachelor’s degree	63 (63.0%)
	Master’s or higher	12 (12.0%)
Experience working in psychiatry unit	Yes	7 (7.0%)
General religious identification	Non-believer	5 (5.0%)
	Believer	90 (90.0%)
	Conservative	5 (5.0%)
Knows someone who is LGB	Yes	21 (21.0%)
Knows someone who is transgender	Yes	7 (7.0%)
Parental attitudes toward LGB individuals	Negative	63 (63.0%)
	Neutral	37 (37.0%)
Parental attitudes toward transgender individuals	Negative	68 (68.0%)
	Neutral	32 (32.0%)
Beliefs about origins of homosexuality	Biological	60 (60.0%)
	Temporary	40 (40.0%)
Beliefs about origins of transgender identity	Biological	61 (61.0%)
	Temporary	39 (39.0%)
Received education on transgender health	Yes	20 (20.0%)
Received institutional training on transgender issues	Yes	4 (4.0%)
Willingness to receive transgender-related education	Yes	64 (64.0%)
Concept: Gender identity	Correct response	83 (83.0%)
Concept: Biological sex	Correct response	83 (83.0%)
Concept: Gender dysphoria	Correct response	39 (39.0%)
Concept: Bisexuality	Correct response	73 (73.0%)
Concept: Heterosexuality	Correct response	87 (87.0%)
Concept: Homosexuality	Correct response	86 (86.0%)
Comfort working with LGB patients	Yes	37 (37.0%)
Comfort working with transgender patients	Yes	38 (38.0%)
Comfort working with intersex patients	Yes	29 (29.0%)
Comfort working with LGBTI colleagues	Yes	33 (33.0%)
**Numerical Variable**	**Mean ± SD**	**Range**
Age	29.86 ± 5.98	20.00–46.00
Years of professional experience	8.04 ± 5.76	0.50–25.00
Transphobia scale score	41.53 ± 12.67	9.00–63.00
Attitudes toward transgender individuals score	57.45 ± 16.42	20.00–96.00
Hudson-Ricketts homophobia scale score	101.06 ± 26.26	12.00–141.00

**Table 2:** Group differences in transphobia and attitudes toward transgender individuals based on sociodemographic, educational, and experiential variables.

Variable	Subgroup	Transphobia (Mean ± SD)	*p* (Transphobia)	Attitudes (Mean ± SD)	*p* (Attitudes)
Gender	Male	39.43 ± 7.41	0.552	56.14 ± 12.51	0.787
	Female	41.69 ± 12.99		57.55 ± 16.72	
Marital status	Single	39.07 ± 11.49	0.132	63.00 ± 15.49	**0.005**
	Married	43.24 ± 13.26		53.59 ± 16.05	
Education level	Bachelor’s degree	43.83 ± 10.81	**0.015**	55.81 ± 15.61	0.113
	High school	35.62 ± 11.59		63.15 ± 18.10	
	Master’s or higher	31.92 ± 14.15		66.33 ± 16.76	
	Associate degree	45.50 ± 15.80		51.00 ± 15.15	
Religious belief	Conservative	40.20 ± 14.96	0.744	55.80 ± 21.43	0.519
	Non-believer	40.20 ± 25.74		68.20 ± 28.49	
	Believer	41.68 ± 11.77		56.94 ± 15.36	
Knows a transgender person	Yes	40.14 ± 15.23	0.695	52.57 ± 20.53	0.690
	No	41.63 ± 12.55		57.82 ± 16.14	
Discusses transgender topics in family	Yes	34.29 ± 13.46	**0.020**	60.41 ± 18.20	0.376
	No	43.01 ± 12.06		56.84 ± 16.08	
Parental attitude toward transgender people	Neutral	36.84 ± 11.26	**0.014**	61.88 ± 15.49	**0.047**
	Negative	43.74 ± 12.77		55.37 ± 16.54	
Beliefs about transgender identity	Biological	39.87 ± 12.00	0.076	60.61 ± 17.56	**0.019**
	Environmental/Temporary	44.13 ± 13.40		52.51 ± 13.20	
Received education on transgender health	Yes	33.95 ± 10.55	**0.002**	70.60 ± 16.02	**<0.001**
	No	43.42 ± 12.50		54.16 ± 14.88	
Received institutional training on transgender issues	Yes	52.00 ± 6.48	**0.039**	49.75 ± 16.74	0.460
	No	41.09 ± 12.70		57.77 ± 16.41	
Willing to receive training	Yes	40.30 ± 12.39	0.144	61.23 ± 17.17	**0.002**
	No	43.72 ± 13.04		50.72 ± 12.60	
Comfort with transgender patients	Strongly disagree	44.07 ± 12.76	**0.007**	47.40 ± 10.76	**<0.001**
	Disagree	45.10 ± 10.08		51.50 ± 13.99	
	Neutral	44.46 ± 13.06		55.03 ± 13.26	
	Agree	39.00 ± 10.72		60.26 ± 16.86	
	Strongly agree	31.18 ± 12.98		77.82 ± 16.49	
Comfort with LGBTI colleagues	Strongly disagree	46.38 ± 13.80	**0.001**	46.50 ± 15.77	**<0.001**
	Disagree	45.39 ± 9.46		54.17 ± 14.14	
	Neutral	43.67 ± 13.35		52.39 ± 11.68	
	Agree	36.85 ± 8.96		64.20 ± 12.32	
	Strongly agree	32.00 ± 12.50		77.92 ± 15.77	

Note. *p*-values < 0.05 are considered statistically significant and are highlighted. Only variables with categorical responses are included. Bolded values represent statistically significant results.

**Table 3:** Spearman correlations between key variables.

	Transphobia	Attitudes Toward Transgender Individuals	Homophobia	Age	Comfort with Transgender Patients	Perceived Knowledge	Received Education on Transgender Health	Parental Attitude (Negative)	Knows the Term “Gender Dysphoria”	Knowledge score
Transphobia		−0.54 **	0.55 **	0.15	−0.35 **	−0.02	−0.31 **	0.25 *	−0.00	−0.03
Attitudes toward transgender individuals			−0.65 **	−0.26 **	0.43 **	0.18	0.38 **	−0.20 *	0.05	0.00
Homophobia				0.33 **	−0.42 **	−0.16	−0.21 *	0.20 *	−0.03	−0.11
Age					0.04	0.21 *	0.08	−0.06	0.18	0.14
Comfort with transgender patients						0.43 **	0.23 *	−0.11	0.24 *	0.31 **
Perceived knowledge							0.21 *	−0.24 *	0.26 **	0.28 **
Received education on transgender health								−0.19	0.08	0.16
Parental attitude (negative)									−0.06	−0.08
Knows the term “gender dysphoria”										0.63 **
Knowledge score										

Note. Spearman’s rho coefficients are reported. * *p* < 0.05, ** *p* < 0.01. Bolded values represent statistically significant results.

**Table 4:** Predictors of transphobia scores: multivariate linear regression analysis.

Independent Variables	B	Std. Error	ß	T	sr^2^	*p*
Const	25.29	10.71	0.0	2.361		0.02
Gender (male)	−4.539	3.906	−0.14	−1.162	0.015	0.248
Religiosity level	−3.456	3.326	−0.093	−1.039	0.012	0.301
Perceived knowledge	3.886	1.491	0.245	2.607	0.07	**0.011**
Homophobia score	0.177	0.041	0.394	4.33	0.171	**<0.001**
Comfort with transgender patients	−1.886	0.981	−0.191	−1.922	0.039	**0.058**
Knows a trans individual	−0.392	3.949	−0.014	−0.099	0.0	0.921
Received education on transgender health	6.923	2.628	0.254	2.634	0.071	**0.01**
Parental attitude (negative)	−4.963	2.195	−0.223	−2.261	0.053	**0.026**

Model Summary: F-statistic = 7.026, Model *p* < 0.001, Adjusted R^2^ = 0.327. B: Unstandardized coefficient; Std. Error: Standardized error; ß: Standardized coefficient B; T: T-value; *p*: Significance level; F: F-statistic; R^2^: Adjusted R-squared; sr^2^: Squared semi-partial correlation. Bolded values represent statistically significant results.

**Table 5:** Predictors of attitudes toward transgender individuals: multivariate linear regression analysis.

Independent Variables	B	Std. Error	ß	T	sr^2^	*p*
Constant	78.329	19.767		3.963		<0.001
Age	−0.331	0.246	−0.121	−1.347	0.021	0.181
Gender	−1.962	4.647	−0.031	−0.422	0.001	0.674
Marital status	1.469	2.803	0.044	0.524	0.004	0.602
Knows a trans individual	6.266	4.634	0.098	1.352	0.021	0.180
Religiosity level	−0.500	4.087	−0.010	−0.122	0.0	0.903
Received education on transgender health	7.976	3.246	0.195	2.457	0.07	**0.016**
Perceived knowledge	0.477	1.854	0.022	0.257	0.001	0.798
Homophobia score	−0.153	0.057	−0.245	−2.686	0.084	**0.009**
Knowledge score	−1.352	0.943	−0.109	−1.433	0.027	0.155
Comfort with transgender patients	2.153	1.959	0.156	1.099	0.016	0.275
Comfort with LGBTI+ colleagues	1.941	1.805	0.147	1.075	0.01	0.285
Parental attitude (negative)	−0.705	2.657	−0.020	−0.265	0.001	0.792
Transphobia score	−0.358	0.117	−0.277	−3.075	0.093	**0.003**

Model summary: F-statistic = 10.14, Model *p* < 0.001, Adjusted R^2^ = 0.526. B: Unstandardized regression coefficient; Std. Error: Standard error; ß: Standardized regression coefficient; T: T-statistic; *p*: Significance level; CI: Confidence interval. *p* < 0.05 values are considered statistically significant and are presented in bold in the table.

## Data Availability

The data supporting the findings of this study are available from the corresponding author upon reasonable request.
